# An Assessment of the Relationships between Extreme Weather Events, Vulnerability, and the Impacts on Human Wellbeing in Latin America

**DOI:** 10.3390/ijerph15091802

**Published:** 2018-08-21

**Authors:** Gustavo J. Nagy, Walter Leal Filho, Ulisses M. Azeiteiro, Johanna Heimfarth, José E. Verocai, Chunlan Li

**Affiliations:** 1 Instituto de Ecología y Ciencias Ambientales (IECA), Facultad de Ciencias, Universidad de la República, Montevideo 11400, Uruguay; gnagy@fcien.edu.uy (G.J.N.); otolito@fcien.edu.uy (J.E.V.); 2 School of Science and the Environment, Manchester Metropolitan University, Chester Street, Manchester M1 5GD, UK; 3 Sustainable Development and Climate Change Management (FTZ-NK), Research and Transfer Centre (FTZ-NK), Faculty of Life Science, Hamburg University of Applied Sciences (HAW), Ulmenliet 20, D-21033 Hamburg, Germany; Johanna.Heimfarth@haw-hamburg.de (J.H.); 15598022233@163.com (C.L.); 4 Department of Biology & CESAM Centre for Environmental and Marine Studies, University of Aveiro, 3810-193 Aveiro, Portugal; ulisses@ua.pt; 5 Key Laboratory of Geographic Information Science (Ministry of Education), East China Normal University, Shanghai 200241, China

**Keywords:** climate disasters, wellbeing, environmental health, climate change, vulnerability, climate change adaptation, socioeconomic determinants, geographical determinants, sustainable development, online survey

## Abstract

Climate change and variability are known to have an influence on human wellbeing in a variety of ways. In Latin America, such forces are especially conspicuous, particularly in respect of extreme climatological, hydrological, and weather events (EWEs) and climate-sensitive disasters (CSDs). Consistent with the need to study further such connections, this paper presents an analysis of some of the vulnerabilities of environmental health issues and climate-related impacts that are focusing on EWEs and CSDs in Latin American countries. The research includes an analysis of the (i) human and socio-economic development; (ii) geographical and socio-economic determinants of vulnerability and adaptability of environmental health issues (exposure, sensitivity, and adaptive capacity); (iii) occurrence of CSDs from 1988 to 2017 and their direct impacts on human wellbeing (Total death and Affected people); (iv) an online survey on the perceptions of the effects of EWEs on human wellbeing in a sample of countries in the region; and (v) discussion of possible solutions. The socio-economic and development indices, and the International Disaster Database (EM-DAT) and Climate-Risk Index (CRI) disaster statistics suggest that the impacts of CSDs are primarily related to socio-economic determinants of human wellbeing and health inequalities. Also, >80% respondents to the survey say that the leading causes of climate-related human impacts are the lack of (i) public awareness; (ii) investment and (iii) preparedness. The paper concludes by adding some suggestions that show how countries in Latin America may better cope with the impacts of Climate-sensitive Disasters.

## 1. Introduction

This paper deals with the direct impacts of extreme climatological, hydrological, and meteorological events, and their associated hazards and disasters (hereafter referred as “Extreme Weather Events” or “EWEs”, and Climate-sensitive Disasters “CSDs” respectively) on human wellbeing, and the life-supporting systems related to environmental health in mainland Latin American countries. The vulnerability issues that are related to human wellbeing, environmental health, and Climate-Sensitive Disasters (hereafter generically referred as “health vulnerability to climate”) are emphasized.

The impacts resulting from sudden shock extreme climate and weather events (EWEs) and Climate-sensitive Disasters (CSDs) on human wellbeing and health are an area which is getting increased attention because the slow-onset changing patterns of climate will bring more frequent and severe EWEs, leading to more burden of disease [1,2,3,4,5]. The increasing evidence of the links between extreme El Niño events and global warming suggest that the occurrence of such uneven EWEs and their associated climate hazards could increase in the future due to climate change [6], which, in turn, is likely to trigger disasters and health vulnerability to climate [7,8,9]. Due to EWEs can be harmful to humans and their life-supporting systems, and even cause loss of lives, managing their risks and disasters is, therefore, crucial when considering its interlinkages with human wellbeing and health [1,2,3,7,9,10,11].

Health is not only an indispensable right but a condition that enables the full enjoyment of other rights. The 2030 Agenda for Sustainable Development and goals (SDGs) reflects this view by proposing a universal, integrated, and indivisible vision that clearly expresses the interlinked nature of human wellbeing and health with economic and environmental sustainability [12].

However, climate change is expected to cause approximately 250,000 additional deaths globally per year from malnutrition, malaria, diarrhoea, and heat stress between 2030 and 2050 [10]. Therefore, in the future, a decrease in public spending on health and social care may aggravate inequality of health outcomes that are related to climate change [12], which in turn will make the region into the most vulnerable area for health [13].

Efforts towards a better understanding of such human impacts have progressed and expanded considerably in the last few years [14,15]. Climate change affects human wellbeing and health in many direct and indirect ways [16,17]. These impacts are primary (direct), secondary, and tertiary (indirect ones). Here, the focus is on the primary impacts (e.g., EWEs) and trauma from CSDs (e.g., wildfires, droughts, and flooding) [18,19]. 

### 1.1. Impacts of Climate Change and Extreme Weather Events on Human Wellbeing in Latin America

The rather low level of human and socio-economic development of most countries in Latin America (LA) makes it one of the most vulnerable regions in the world, as far as the impacts of climate change, EWEs, and CSDs are concerned [19,20,21]. Some of the effects of climate change influencing health in LA are the (i) increased frequency of EWEs; (ii) worsened ambient air quality; (iii) altered distribution of allergens; and (iv) a modified distribution of vector-borne infectious diseases [3,6,10,12,15,19]. Also, the adaptive capacity of the human systems in LA is low when compared with the industrialized countries, particularly against EWEs, and their vulnerability is high [19]. People living in poor households and/or exposed to climate hazards, for example, are particularly vulnerable to the resulting health risks and are exposed longer to the health consequences from slow-onset climate change problems, and particularly, sudden EWEs (e.g., cold and heat stress, droughts, flash floods, crop failure, and sea-level rise), and their related CSDs [12,17,18,19]. For instance, from 2000 to 2015 the disasters in South America have increased significantly affecting almost 74 million people [21]. The most common disasters have been floods (50%), followed by storms (9%), landslides (8%), and extreme temperatures (8%) [22]. This recent worsening of trends has been leading to changes in human mobility, as people tend to abandon some areas due to unfavourable environmental conditions [20,22].

Future climate projections suggest increases in temperature and changes in precipitation for Central America (CA) and South America (SA) by 2100 [21]. LA countries, which contribute little to climate change but are hard-hit by extreme weather events [3,19,23], urgently need new indicators of vulnerability and exposure to EWEs to back up their claims for financial and technical assistance [23].

### 1.2. Defining Key Concepts: Human Wellbeing, Health Vulnerability to Climate, Environmental Health and Climate Change Adaptation

The following interrelated and complementary definitions focus on the research framework of this article.

(1) Human wellbeing is a holistic construct that goes well beyond the dimensions of biophysical health services, e.g., clean air, a safe and adequate water supply, and a global ecosystem that will continue to provide these services at an individual level. Its meaning is captured in the World Health Organization’s definition of health “a state of complete physical, mental and social wellbeing and not merely the absence of disease and infirmity” [24]. These biophysical health services are closely related to the life-supporting systems concept used by the Notre Dame Gain Index [25], as well as to environmental wellbeing.
(2) The vulnerability is composed of three elements: Exposure-E, Sensitivity-S, and Adaptive Capacity-AC [4]. Human health vulnerability to climate is a function of:
  (i) Sensitivity: the extent to which health (or the systems on which health outcomes depend), are sensitive to changes in weather and climate (the exposure-response relationship) and the characteristics of the population; (ii) the exposure to the climate-related hazard (the character, magnitude, and rate of climate variation); and (iii) the adaptation actions that are in place to reduce the burden of a specific adverse health outcome (the adaptation baseline), the effectiveness of which may influence the exposure-response relationship [26].
  Vulnerability measures a country’s exposure, sensitivity, and ability to adapt to the negative impact of climate change in life-supporting services, where E is the nature and degree to which a system is exposed to significant climate change, independent of socioeconomic context, sensitivity is the extent to which a country is dependent upon a sector negatively affected by climate hazards, or the proportion of the population that is particularly susceptible to climate change hazards, and AC is the availability of social resources for sector-specific (current or potential) adaptation capacities. The ND-Gain health score (vulnerability of water, habitat, and health services sectors) captures a country’s of public health vulnerability to climate change, regarding the spread of communicable diseases and provision of health services [25].
(3) Environmental health is the science and practice of preventing human injury and illness and promoting wellbeing by (i) identifying and evaluating environmental sources and hazardous agents and (ii) limiting exposures to hazardous physical, chemical, and biological agents in air, water, soil, food, and other environmental media or settings that may adversely affect human health [27].
(4) Climate Change Adaptation (CCA) refers to “the process of adjustment to actual or expected climate and its effects” [4].

### 1.3. Research Framework

There are unfortunately few international studies that address the climate disasters-human wellbeing-sustainable development nexus in the Latin American (LA) context. This article aimed to make a cross-comparison survey that looked at a set of seventeen mainland (LA) countries from Central (CA), Mexico (Mx), and South America (SA) about the health vulnerability and direct impacts on human wellbeing that are associated with (i) (EWEs) and (ii) (CSDs), based upon the disaster country-level statistics (EM-DAT database from 1988 to 2017, and Global Climate-Risk Index (CRI) from 1997 to 2016, see Section 2). Additionally, an online survey with regional environmental and climate researchers supports the disaster statistics. The study emphasizes primary human impacts and stress of EWEs, namely total death and affected people.

The working hypothesis is, as modified from Leal Filho et al. [17]: “All countries and regions worldwide show some degree of vulnerability, are exposed and are directly or indirectly impacted by climate-related hazards regardless of their socioeconomic status and readiness”.

The research problem is related to the following question based on the working hypothesis: “Are the direct human impacts of CSDs primarily due to their (probably increased) frequency and intensity, or to the intrinsic human and socio-economic determinants of wellbeing and health inequalities?”

Therefore, the objectives of this paper are to respond to these questions. For doing so, an update of relevant indices that are related to the human vulnerability to climate and the impacts of extreme climate/weather events on human wellbeing from 1988 to 2017 is carried out. Some suggestions, which are based on these results, the literature, and an online survey, are made, about how LA countries could improve climate change adaptation (CCA) of the public health sector to CSDs and their link with disaster risk reduction (DRR).

**Figure 1:**
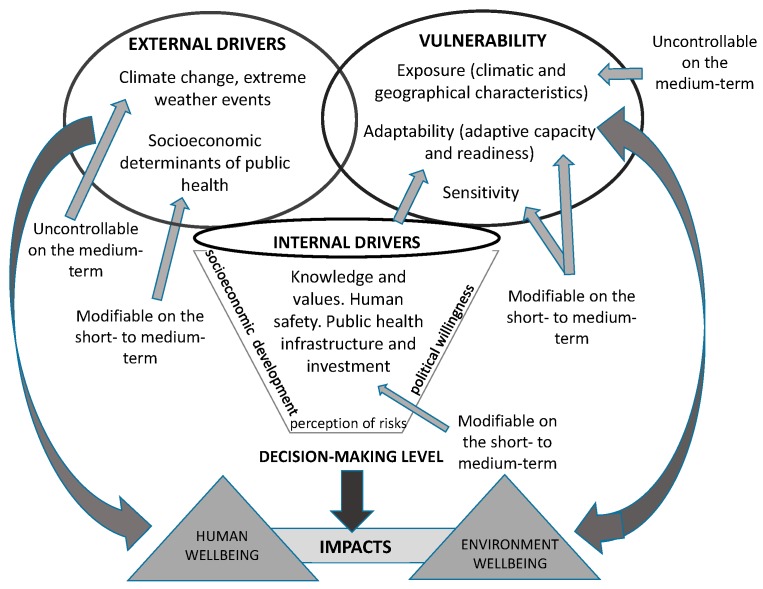
Drivers-Vulnerability-Actions interactions schematic (inspired by [1,4,17,25,26]).

The basic concepts, terms, and assumptions of the analytical framework that is used to construct causal explanations [28] of the Drivers-Vulnerability-Actions interactions (Figure 1) causing the observed Climate-Sensitive Disasters and their impacts on human wellbeing to be as follows (based on [1,17,25,26]):

(i) External climate drivers (threats) are (assumed) uncontrollable on the short- and medium-term (e.g., 2025–2040) at the local level, whereas socioeconomic driving determinants are (considered) (almost) to be uncontrollable on the short-term, but potentially modifiable [17];
(ii) The drivers of this system are external: climate change and extreme weather events (assumed uncontrollable on the medium-term), and socio-economic determinants of public health (supposed modifiable on the short- to medium-term); internal: knowledge and values; human safety, public health infrastructure, and investment (assumed as controllable on the short-term but dependent on the level of socioeconomic development, perception of risks, and political willingness);
(iii) The vulnerability is composed by (1) the exposure (climatic and geographical characteristics, e.g., climate type/sub-types, location, and population density) [1,25], assumed as being uncontrollable on the short- to medium-term, (2) the sensitivity (e.g., % of population affected by climate-hazards and their susceptibility [1,25,26]), and (3) the adaptability composed of the adaptive capacity and readiness [25]. The components ii and iii are moderately modifiable on the medium-term;
(iv) The actions relate to the country-level decision-making level (anticipatory climate adaptation) that are both influenced by and influence the concepts and terms mentioned above. Measures that are not primarily intended to reduce the current climate vulnerability and socioeconomic determinants are close to disaster preparedness and response, such as weather forecasting, early warning systems, evacuation, and relief (assumed as modifiable on the short-term).

All of these elements overlap within a continuum interacting system. For instance, readiness, preparedness, and response are closely inter-related. The former is Climate Change Adaptation (CCA) and the latter is Disaster Risk Reduction (DRR), which, ideally, should be regarded as inter-linked [5].

Some of the principal expected outputs of this research are as follows:

(i) extreme weather events directly affect the human wellbeing of vulnerable people, particularly among the poor and those more exposed people, and influence public health policies;
(ii) wellbeing and health impacts of extreme weather events are linked to the human and socioeconomic development determinants of the health inequality, and the expenditures in Public Health; and,
(iii) building resilience to extreme weather events and policies to support it, linked with disaster risk reduction actions are needed.

## 2. Materials and Methods 

The methodology of this study consisted of four steps:

(i) Step 1: Identification of the sample of countries and compilation of a list of climate-stressors, geographical, human, and socioeconomic determinants of the health vulnerability and safety issues, extreme weather events (EWEs), and Climate-Sensitive Disasters (CSDs) in the studied countries;
(ii) Step 2: Collection of evidence-based statistics of EWEs and CSDs from 1988 to 2017 and their direct human impacts from the public available source EM-DAT CRED database [29] and the Global Climate-Risk Index-CRI from 1997 to 2016 [30];
(iii) Step 3: Collection of opinions from a sample of LA climate and environmental researchers and practitioners about climate change, EWEs, health vulnerability to climate, and the main perceived barriers and possible solutions in LA; and,
(iv) Step 4: A comparative qualitative and quantitative analysis of the geographic and socioeconomic determinants of the vulnerability in regards to EWEs, CSDs, and wellbeing and environmental health.

The study addresses the following methodological processes:

### 2.1. Identification of the Sample of Countries and Compilation of the Primary Determinants of Health Vulnerability to Climate and Adaptation to Extreme Weather Events (EWEs)

The collection of the vulnerability, risk, and adaptation issues was made from the sample of seventeen mainland countries of Latin America, as follows: South America (SA: N = 10), Central America (CA: N = 6), and Mexico (Mx). The LA Caribbean islands will be subject to another study by the International Climate Change Information Programme (ICCIP).

The vulnerability is different from risk. Whereas, risk is about exposure to external hazards over which people have limited control, vulnerability is a measure of people’s capacity to manage such hazards—to prepare for, cope with, and recover from them without long-term, potentially irreversible losses of wellbeing [31].

Geographical factors, such as land area and populations, serve to assess the impacts normalised by the Population Density. Decreasing/increasing values order human and socioeconomic indicators expressed as per capita or percentage (%), absolute figures, and discrete classes (1–5).

The indices used are:

(i) the Notre Dame University Gain Index [25];
(ii) the German Watch Global Climate-Risk Index (CRI) [27];
(iii) the United Nations Human Development Index (HDI) [32]; and,
(iv) the Legatum Prosperity Index for Health (LPHI) [33].

The ND-GAIN Country Index (ND-VR) summarises a country’s vulnerability to climate change and other global challenges in combination with its readiness to improve resilience. The ND-VR measures vulnerability (Exposure + Sensitivity − Adaptive Capacity) and readiness (a country’s ability to leverage investments and convert them into adaptation actions) when it comes to climate change and weather-related hazards impacts (CSDs due to EWEs).The specific analyzed sectors that are related to the health vulnerability to climate are (i) Public health (PHV): The spread of communicable diseases and provision of health services; (ii) water (WV): Freshwater supplies and access to reliable drinking water; and (iii) Human habitat (HHV): Human living conditions, while considering weather extremes. Exposure (E) relates here to the geographical location and the occurrence of climate changes and hazards (Independently of socio-economic context). This definition of exposure is very similar to the one that is given for risk.

Generally, a composite index is developed to either measure a multidimensional concept or to describe a system. In cases where the goal is to measure a multifaceted concept, an aggregation of a parsimonious set of indicators can be effective [34]. Here, the additive unweighted ND-Gain sectoral health vulnerability indices [∑V = PHV + WV + HHV], exposure and sensitivity [E + S], and Adaptability indices Adaptive Capacity (AC) and Readiness (R) [AC + R] were classed by the authors (1, 1.5, 2.0…, 4.5, 5 scales) based on their distribution in the ND-VR ranked tables (classed initially with colours in the ND-Gain tables)so as 1, 2…, 5 represent the quintiles of the distribution. There can be no claim that this additive aggregation is an index, but a way to rank and compare countries by a set of indicators. Then a relative rank (among the seventeen LA countries) was made for these two blocks of aggregated indicators (Vulnerability—CRV and Adaptability—CRA).

The HDI, for example, organizes indicators into three main dimensions of human wellbeing: health, education, and income [32]. These socio-economic and human development indicators are (i) the per capita parity purchase power (PPP) Gross domestic product (GDP); (ii) Education level of the population; and (iii) Life Expectancy (health status). This index is classed (1–5 quintiles) as very low, low, medium, high, and very high [32]. HDI is a standard index in studies on the nexus climate adaptation, extreme weather events, sustainable development, and DRR [17,35,36,37,38].

The CRI analyses to what extent countries have been affected by the impacts of weather-related events (EWEs) from 1997 to 2016 [27]. The Legatum Prosperity Index (LPI) offers a unique insight into how prosperity is forming and changing the world. The LPI ranking is composed of nine equally-weighted sub-indices, one of which is health (LPHI). This sub-index measures a country’s performance in basic health, health infrastructure, and preventative care indicators [33].

The Per capita Gross National Income GNI = (GDP + net income received from overseas − income earned in the domestic economy by non-residents) [39].

Poverty expressed as the percentage of the population [40] is a direct indicator of human and socioeconomic status, health inequality, and an indirect sign of social vulnerability against climate hazards. Poverty and vulnerability are interrelated as they influence each other and as very often poor people are the most vulnerable to any adverse effects of any risk (climatic or socioeconomic) [41].

### 2.2. Extreme Climate and Weather Events and Climate-Sensitive Disaster Statistics (1988–2017) in the Seventeen Studied LA Countries

An analysis of climatological, hydrological, and meteorological disasters (CSDs) was carried out based on the annual country-level statistics from 1988 to 2017 for floods, drought, storms, landslides, and wildfires from the EM-DAT CRED database [29]. The accumulated occurrence of all disasters (Total Entries) was 1096 (456 from CA and Mx, and 640 from SA). EM-DAT continuously updates the country-level Top Ten disasters (and their impacts), which are also presented (as of the end of 2017).

### 2.3. A Survey on Perceptions of the Health Impacts of Extreme Weather Events and Sea Level Rise in Latin America

Bearing in mind the limitations and constraints seen in data collection as part of international studies, the method that is chosen to complement the selected indices and statistics at the national level, was an online survey, deployed to gather information from a variety of Latin American researchers and stakeholders.

The online survey was disseminated via email (in two calls, 30 days apart from each other) from November to December 2017 while using Google Forms. The sample was based on those who took part in the survey out of their own will. Consistent with good academic practice and based on the need to adhere to data protection procedures, the study was anonymous.

The survey instrument was composed of 14 questions (12 closed questions and two open questions) that aimed to characterize the perceptions on climate change, risk, and extreme events impacts, on human wellbeing and health in mainland LA countries. The Supplementary Information includes a copy of the online survey (Table S1: Online Survey Responses).

A total of 52 responses were received, checked, and analyzed for percentages and frequencies on the closed questions data collected, and content (categories) of open items, and subsequently quantified as percentages.

The survey had the following limitations: Firstly, a relatively small sample (52 responses) when compared to other studies, partly because the study was performed with no external support. Secondly, there is some geographical imbalance in the answers to the survey (e.g., countries underrepresented). Thirdly, the time scale of only one month. Because of these limitations, the reliability of the data is limited and it is not statistically representative of independent research. However, since scientists that were working on climate-related human impacts research provided the questions, and respondents volunteered to submit their contributions since the processing of the data was transparent and since the discussion of the manuscript acknowledges and keeps in mind the limitations of the survey, it is only an additional input that is used to support the debate. Therefore, the data collected allow for only a rough profile of the current public perception of the extreme events, preparedness, and barriers to adaptation, and a few suggestions to reduce vulnerabilities.

### 2.4. Comparative Analysis of the Determinants of EWEs, CSDs, and Environmental Health

To describe the qualitative and some quantitative relationships between the climate-risk, and human and socio-economic indices of development, vulnerability, and adaptation, the indicators and indices were not weighted. However, per capita GDP appears in more than one aggregated index (e.g., HDI), which indirectly enhances its weight. 

Regression and correlation analysis (R-Pearson: RP or R-Spearman: RS, with their levels of significance *p* < 0.05 and *p* < 0.01) were performed between the climate-risk, geographical, human, socio-economic, and health vulnerability/adaptation indicators/indices with the figures of impacts due to CSDs, and the most relevant and significant ones were retained. All data were ordered and ranked. The ND—Gain vulnerability and adaptability indicators were discretised in five classes (1–5) or quintiles from the original data.

The indices that are used for comparative analysis (e.g., regression, R-Pearson, or R-Spearman) are the (i) HDI; (ii) Poverty (% of population); (iii) GNI, LPHI; (v) Fatalities (per Million inhabitants) from the CRI, and the aggregated blocks of vulnerability sectors that are related to human wellbeing (water, public health services, habitat), plus exposure (E), Sensitivity (S), and adaptability (AC + Readiness). All of these were ranked (among the 17 countries), and some were classed to illustrate semi-quantitative relationships (e.g., classed ranked sectoral vulnerability, E and S, and classed ranked adaptability-CRA).

## 3. Results

This section is divided into four subsections, as follows.

### 3.1. Geographical, Socio-Economic and Vulnerability Setting

This section presents country-level indicators and indices of human and socio-economic determinants of health vulnerability to climate, risks, adaptation, and direct impacts on human wellbeing due to extreme weather events (EWEs) and climate-sensitive disasters (CSDs) from EM-DAT and CRI databases. Some quantitative relationships between indices and human impacts (e.g., Fatalities per Million Inhabitants) were carried out. This subsection is divided as follows.

#### 3.1.1. The Geographical and Climatic Setting of Extreme Weather Events (EWEs)

Table 1, Table 2 and Table 3 summarise the land area and populations [42], the prevailing Köppen classification climate-types [43], and the main weather extreme events [27] of the studied countries.

The prevailing Köppen climate-types are the following: (i) Tropical (A type: 40%), which prevails in Brazil, Colombia, Ecuador, Venezuela, and CA; (ii) Temperate (C: 39%), which prevails in Argentina, Chile, Paraguay, Peru, and Uruguay, and semi-arid (B type: 21%), which prevails in Bolivia and Mexico. The most common subtypes are Tropical Wet-AW (19%), mostly in CA, northern SA countries, and Brazil, and Temperate Oceanic-Cfb (16%), in the coast of Peru as well as in most coastal states in SA and CA.

The prevailing EWEs are Riverine floods (in ten countries), storms (in four countries), and droughts (in three countries). Extreme (ext.) temperatures (mostly cold ones) are typical in at least seven countries.

#### 3.1.2. Human and Socio-Economic Development, and Health Vulnerability Status

Table 2 summarises the human and socio-economic development status, and the ND-Gain vulnerability (exposure, sensitivity, adaptive capacity) sectoral indicators that are related to wellbeing and health [(PHV, WV, HHV) + E + S] = [∑V + E + S]], and adaptability [AC + R] are summarized. The classed (1–5) vulnerability and adaptability (CRV and CRA, respectively) were discretised by the authors from quintiles distribution of the ND-Gain data.

In Table 3, the GDP Per Capita (in current U.S. $), and the public and private expenditures in Health are presented based on data from the Pan American Health Organization-PAHO/WHO [18]. The authors calculated regional ranks and per capita expenditures from these data. 

#### 3.1.3. Descriptors of Extreme Weather Events

This section describes the descriptors of the fatalities due to EWEs in the studied LA countries from the datasets that are provided by the CRI statistics (1997–2016), ND-Gain LPHI Indices, and EM-DAT statistics (Number of EWEs, regional rank, and frequency per year for time horizons A: 1988–2017 and B: 1997–2016, respectively, so as to estimate a trend from A to B: increase ↗, decrease ↘ and similar ≈). Geographical Exposure was determined based on the population density (PD) (Very Low: 1 to Very High: 5) through normalisation per population and area, and classed as 1: very low and 5 very high, where the lowest the best (fewer climate-risks and fatalities). Table 4 summarises the data used for the analysis, including the GNI, HDI, and LPHI indices.

#### 3.1.4. A Few Selected Quantitative Relationships between Indices and EWEs

**Figure 2:**
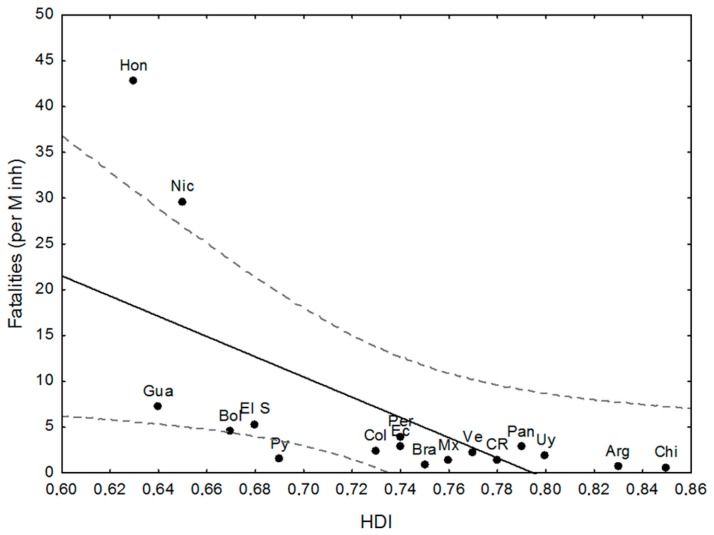
Human Development Index (HDI) vs. Fatalities (per M inh.) in the studied countries. Source: Climate-Risk Index (CRI) Report [30].

Figure 2 shows the relationship between the HDI 2016 and the number of fatalities (Fat) per Million inhabitants (M inh.) in the studied countries (see Table 2 and Table 4). Nicaragua, Honduras, and Paraguay deviate from the linear function HDI-Fat (R_P_ = −0.635, *p* < 0.01; without outliers, R_P_ = −0.872, *p* < 0.01). The eight countries with HDI ≥ 0.75 have fatalities ≤3 per M inh., as well as Paraguay, which shows fewer impacts than expected from their development status.

**Figure 3:**
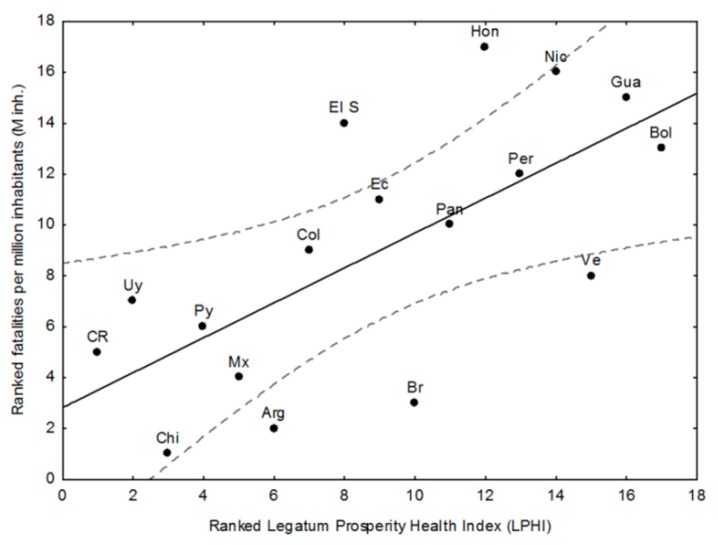
Ranked Legatum Prosperity Health Index (LPHI) vs. the ranked fatalities per million inhabitants (M inh.) in the studied countries.

If the ranked LPHI 2017 (see Table 4) is used as an independent variable against the ranked fatalities (Figure 3), a positive linear relationship is found (Rs = 0.69, *p* < 0.01) with Argentina, Brazil, and Venezuela performing better than expected, and El Salvador and Honduras performing worse than expected from this LPHI descriptor.

**Figure 4:**
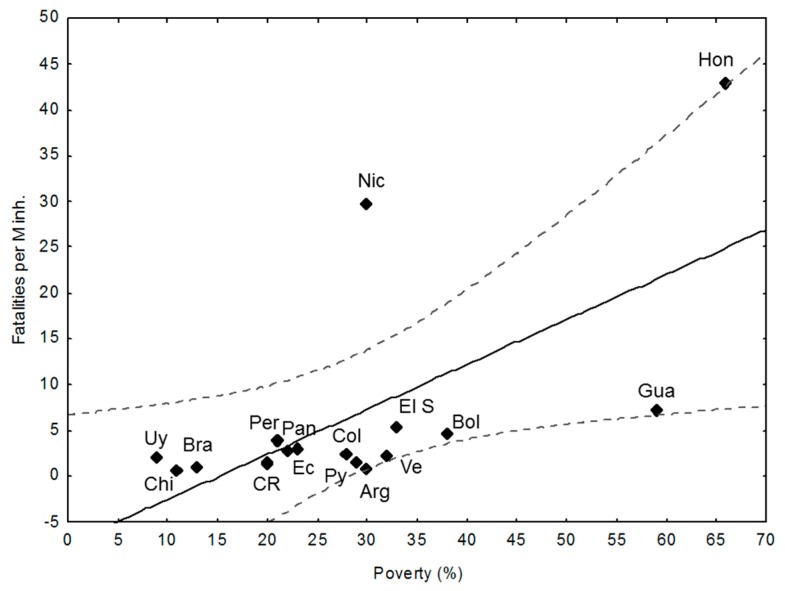
Poverty (%) vs. Fat. Per M inh.

The relationship poverty rate (%) (see Table 2) vs. fatalities per M inh. (Figure 4) is linear (R_P_: 0.64, *p* < 0.01) with Nicaragua as an outlier.

The population density shows a moderate correlation with fatalities per M. people (R_P_: 0.55, *p* < 0.05, not shown) for 15 countries (except for the outliers Honduras and Nicaragua biased by the occurrence of Hurricane Mitch in 1998); the states with the highest value are El Salvador and Guatemala.

The recent trend of occurrence of EWEs (1997–2016) is increasing (see Table 4) in at least five countries (Argentina, Bolivia, Chile, Uruguay, and Panamá), whereas it decreased in Honduras (after Mitch).

Table 5 shows the country-level top-ten natural disasters, the number and type of climate/weather ones among them, and the Total Affected Population (TAP in Million People). The countries with the highest percentage of TAP due to natural disasters are Honduras, Nicaragua, Peru, Costa Rica, Bolivia, and Brazil (67%, 45%, 38%, 26%, 25% and 24%, respectively), while those with the lowest percentage are Venezuela, Ecuador, Chile, Uruguay, and Mexico (3%, 4%, 5%, 5% and 8%, respectively). In most countries EWEs prevail among the top-ten natural disasters, reaching 10/10 in Argentina, Bolivia, Honduras, Mexico, Paraguay, and Uruguay. 

**Figure 5:**
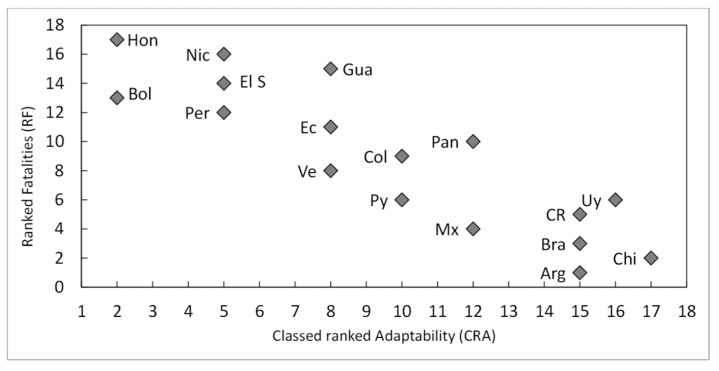
Classed ranked Adaptability (CRA) vs. Ranked fatalities (RF).

The classed aggregated ranked vulnerability-CRV (related to human wellbeing and health sectors, exposure, and sensitivity), is linearly related with the adaptability (CRA) (Table 2) (not shown here), which shows a good fit with the country-level ranked fatalities (RF) (per million inh.) (Figure 5). Given that CRA was not tested (as an index), this relationship is only used to illustrate the importance of readiness and adaptation.

Focusing on the relationships between the chosen descriptors and those that show better/worse performance than expected from the descriptor and the outliers (regarding the confidence limit *p* < 0.05), some patterns emerge from Figure 2, Figure 3, Figure 4 and Figure 5 and Table 1, Table 2, Table 3, Table 4 and Table 5:

- there is a negative and significant relationship between HDI and the number of fatalities, including the outliers (high figures of Nicaragua and Honduras, and the low ones of Paraguay);
- Paraguay performs better than expected from HDI and in line with its good LPHI (ranked 4th, see Table 4), while Argentina, Brazil, and Mexico perform better than expected regarding their HDI, and Brazil and Argentina do the same regarding their LPHI;
- there is a positive and significant linear trend between poverty (%) and fatalities per M inh. (r: 0.64, *p* < 0.01). Argentina, Paraguay, and Guatemala perform better than expected from this trend;
- the high and very high poverty percentages of Nicaragua (30%), El Salvador (33%), Bolivia (38%), Guatemala (59%), and Honduras (66%) are quite well correlated with fatalities, while Argentina (30%) and Paraguay (29%) perform better than expected from the trend, which is in line with their LPHI. Guatemala’s fatalities lie below expected from HDI, poverty, and LPHI;
- the HDI index, the poverty rate (%), and the classed LPHI and CRV are quite well related to the fatalities per M inh. (and the RF), except for outliers Honduras and Nicaragua, which show terrible socio-economic and vulnerability indicators. There are a few examples of countries performing somewhat better (which does not mean well) than expected from the chosen descriptors (e.g., Argentina and Paraguay); and,
- the aggregated discrete adaptability (CRA) shows a good fit with the ranked fatalities (RF). The same, but less clear was for CRV (not shown here).

### 3.2. Online Survey: Perceptions of the impacts of Climatic Changes and Extreme Weather Events (EWEs) on Human Wellbeing and Health in LA Countries, and Possible Solutions

An online survey on the perceptions of the impacts of Climate Changes and EWEs on human wellbeing and health in LA countries and possible solutions is presented to support the CSD statistics, and to further discuss its linking with data.

The number of respondents to the questionnaire was 52, most of them from the academia (74%), 75% of which came from Argentina, Brazil, Mexico, Paraguay, Uruguay, and Venezuela; although the survey data is small, it contributes somehow to respond to the research problem. The Most common extreme events that were identified by the respondents were as follows (Table 6):

(i) inundation and river floods (94%);
(ii) storms (76%, 18% of which coastal storm surges);
(iii) droughts (67%);
(iv) fires (48%);
(v) heat waves (47%); and,
(vi) others (23%, e.g., hurricanes, sea-level rise, hail, landslides).

Two selected excerpts from the participants are.

(i) “The preliminary draft of the Framework Law on Climate Change, drafted for just over a year, is in the process of socialisation, seeking to put Paraguay among the first countries to address this problem at the global level”.
(ii) “The XXI century needs the integration of self-care, care for the other, and care for the whole planetary ecosystem. There is a need for a new ethos (neoethos) in our health professions; we need to transform our paradigms of health”.

### 3.3. Linking Data to Perceptions

Here, the results presented in Section 3.1 and the perceptions introduced in Section 3.2 are compared. Most common extreme events that were identified by the respondents to their country are in line with data presented in Section 1.1: Floods (50%), storms (9%), landslides (8%), and extreme temperatures (8%), and Table 1, Table 3 and Table 4: Floods followed by storms and droughts (likely biased by the origin of the respondents, mainly from flood-prone countries instead of storm-prone nations). 

When the topic is climate change information the references to the media are immediate and when asked “what could be done” respondents also mention “Information and Media”.

Preparedness in the region is negatively perceived and concomitantly when asked “what could be done” respondents prioritise “Information systems in a changing climate, Early Warning Systems, Risk Assessment, management and communication, preparedness”. The above statements also highlight the importance of extreme events (Table 4) in a context of high vulnerability (Table 2) and geographical exposure (Table 1 and Table 4).

There is an observed high level of awareness of the links between climate change and human wellbeing and health. Climate change, environmental health, health inequalities, assumes greater importance in regions where poverty (Table 2) poses a challenge to social justice, environmental justice, and sustainability (health risks and access to health resources). When asked “what could be done” respondents prioritise “Health capacities, Promoting Health, epidemiology of the identified priority vector-borne zoonotic diseases” but also “Capacity Building and Partnerships”, and also mention “Participation, empowering stakeholders to build partnerships, participate in building resilience in a changing climate”.

### 3.4. Classed Ranked Vulnerability and Adaptability, and Climate-Risk

**Figure 6:**
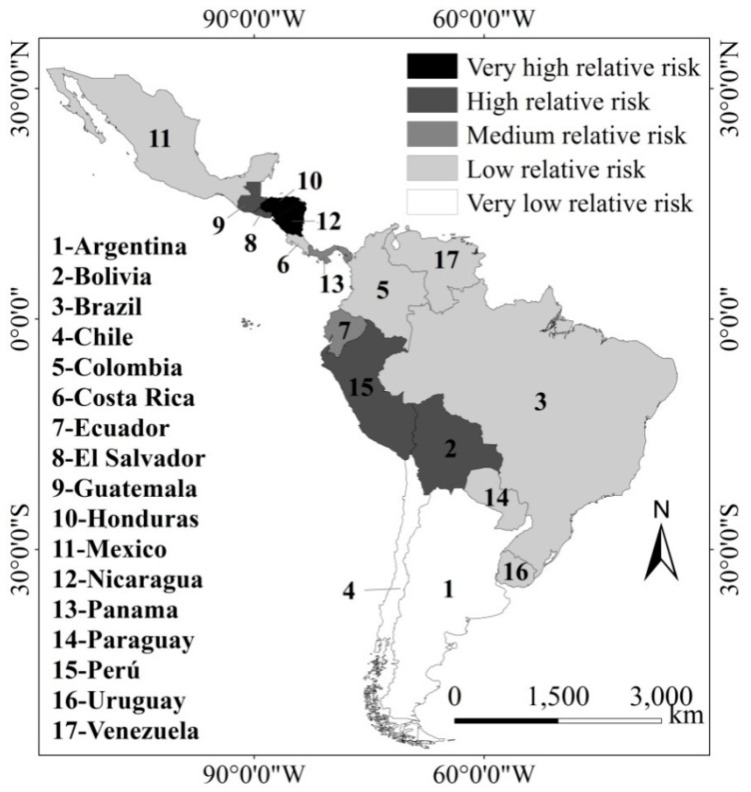
Classed relative risk to extreme weather events of the studied countries (estimated by the authors from the classed ranked Vulnerability and Adaptability).

Based on the classed ranked vulnerability and adaptability (Table 2, Figure 4) and the Total Affected People due to Top-ten EWEs (Table 5), the authors have compared the countries’ relative risk (Figure 6) from very low (white) to very high (black) to Climate-sensitive Disasters. For instance, the extremes include Honduras and Nicaragua (very high relative-risk) and Argentina and Chile (very low relative-risk).

## 4. Discussion

This article discusses the occurrence and direct human wellbeing impacts of climate variability and extreme events in mainland Latin American countries that were centred over the period 1988–2017. The principal assumptions are as follows: (i) an increase in the frequency and/or magnitude of Extreme Weather Events [1,2,3,4,5,6,12,19,20,21,22,23]; (ii) all countries show some degree of vulnerability, are exposed, and are impacted by EWEs, regardless of their socio-economic status and readiness [17]; and (iii) the geographical and climatic settings are mostly uncontrollable, while the socioeconomic determinants of health vulnerability to climate are assumed modifiable in the medium-term. 

### 4.1. The Assessment of the Determinants of Vulnerability and Impacts

The assessment builds on the geographical and socio-economic determinants of health vulnerability to climate and human wellbeing impacts from several indices, and the International Disaster Databases EM-DAT and Global Climate Risk Index-CRI (Table 1 and Table 4). Over the studied period (1988–2017), the most conspicuous EWEs have been(i) riverine floods, (ii) storms, and (iii) droughts, followed by extreme temperatures, wildfires, and landslides, which agrees with other previous studies [19,23,44].

The following socio-economic determinants of health are relevant in this discussion:

(i) Inequity “A disparity in health outcomes that is systematic, avoidable, and unjust” [45];
(ii) Inequality “Differences, variations, and disparities in the health achievements of individuals and groups of people” [46];
(iii) Disparity “A type of difference in health that is closely linked with a social or economic disadvantage that negatively affects groups of people who have systematically experienced greater socioeconomic obstacles to health (e.g., socioeconomic status, geographic location)” [47].

These concepts relate to the determinants of health (e.g., human development, physical environment, social structures, economic systems, health services) [48] that determine the impossibility to afford basic needs for human wellbeing, such as clean water, nutrition, healthcare, education, clothing, and shelter. 

Climate events worsen health disparities in developing countries as a whole and weather extremes in particular. As a result, environmental health problems that are associated with the indirect climate impacts [16], such as stress due, for instance, to damages to crops and properties, thermal discomfort [49], or increases in the transmission of mosquito-borne diseases [50] tend to widen the gap and make populations in developing countries even more vulnerable than they already are. Equality stands at the centre of development; the gaps in the provision of services are associated with the persistence of slums, whose inhabitants frequently face higher risks of exposure to communicable diseases, environmental pollution, and natural disasters [51].

The disparities in the wellbeing and health geographical, climatic and socioeconomic determinants presented in Table 1, Table 2, Table 3, Table 4 and Table 5 are mutually-aiding with the CSD-related human impacts. They provide qualitative and quantitative insight to answer the research question ”Are the direct human impacts of CSDs primarily due to their (probably increased) frequency and intensity or to intrinsic human and socio-economic determinants of wellbeing and health inequalities?”.

The geographical and climatic determinants (Table 1) are a significant and fundamentally uncontrollable factor of country-level health vulnerability to climate. For instance, (i) most storm-prone countries have a tropical climate, mainly in CA; and (ii) most riverine flood-countries are temperate, subtropical SA countries. The smallest and highly populated CA countries, notably El Salvador, Honduras, and Nicaragua, show high human impacts beyond other main (modifiable) determinants. 

The development status, vulnerability, adaptation and climate-risk indices (Table 2, Table 3 and Table 4), and health expenditures (Table 3) show strong country-level disparities. For instance, (i) poverty levels vary from only 9–11% (Uruguay and Chile, respectively) to 59%–66% (Guatemala and Honduras respectively), and the Climate Risk Index and fatalities values range from very low (Argentina, Brazil, Chile, and Costa Rica) or low (Panama and Uruguay) to very high ones (El Salvador, Guatemala, Nicaragua, and Honduras). The aggregated indices HDI and LPHI are quite well correlated with the number of fatalities per million inhabitants, while poverty level, a proxy of social vulnerability [41], is likely a key determinant (Figure 3), without overlooking the exposure to and the magnitude of EWEs. 

The Classed Ranked Adaptability (CRA) aggregated by the authors (Table 2) from the ND-Gain indices is inversely related to the country-level ranked fatalities (RF), highlighting the importance of the human, socioeconomic and political willingness determinants of the capacities to implement adaptation measures (see Figure 1) and the ability to leverage investments and convert them into adaptation actions (readiness). For instance, the Total Health Expenditures (HE) vary from 4.8% (Argentina) to 9.8% (Paraguay) (% of GDP) and from US$ 175 in Nicaragua to US$ 1352 in Uruguay (Per Capita GDP in US$). The ranked fatalities per M inh. are well correlated with the ranked HE in most (N: 13) countries (Rp: 0.63, *p* < 0.01, not shown here). The lousy performance of Guatemala coincides with the worst social determinants, e.g., the (i) very low public HE (2.3%) and GDP (ranked 14th), high poverty (59%), besides high population density and climate-risk index.

Are the relative high expenditures in the health of Paraguay a cause of the below-expected fatalities in this country? The excerpts taken from the online survey suggest the existence of a high level of awareness, which could be a consequence of the relatively high percentage of people affected by CSDs (see Table 5), which is mainly due to extreme floods [52,53].

The Total Affected People (TAP) indicator (Table 5) shows a high diversity with countries with very low TAP due to CSDs (Chile, Ecuador, Uruguay, and Venezuela) to very high figures (Guatemala, Honduras, Nicaragua and Peru). The exposure of small storm-prone CA countries (e.g., Guatemala and particularly Honduras and Nicaragua) could be explained by their high population density, prevailing climate, and disaster profile, besides low to very low adaptability and health expenditure indices. On the other hand, the case of Uruguay (TAP = 5%) is particularly interesting because it is a flood-prone country (like Argentina and Paraguay) and it suffers frequent extreme windstorms. Its relatively low vulnerability may be attributed to its good Adaptability, as shown in Table 3, and to repeated evacuations of the same people along the flood-prone Uruguay River [53].

An example of a successfully anticipatory adaptation measure linked with Disaster Risk Reduction is the Early Warning System, and vulnerability, hazard, and risk map for extreme rainfalls and landslides of La Paz City. The integrated climate and weather forecasting and the risk-mapping system were developed after a deadly landslide occurred in February 2002 with a death toll of 63 persons plus 14 missing persons, in addition to the losses and damages to habitat, roads, and water supply and sanitation services. In February 2011 another extreme rainfall occurred, which activated the Early Warning and Risk-mapping System (EWRMS), allowing for prompting the evacuation of inhabitants at risk, which prevented the loss of human life due to the landslide. The first preparedness and response differences with the event of 2002 were: (i) the communication did not fail (in 2002 phone cell lines were impacted); and (ii) the existence of an operational EWRMS [54].

Finally, it is worth mentioning in regards to future adaptation that the studied countries, except Brazil, have not implemented National assessment of climate change for health, nor have they taken measures to increase the climate resilience of health infrastructure yet [2].

### 4.2. Main Contributions to the Analysis Extracted from the Online Survey

The online survey (Table 6) provides several responses regarding EWEs, barriers, policy actions, and approaches to health policy and the Nexus Environment-Human Wellbeing under changing climate-risks.

The perception of main EWEs is similar to other studies (e.g., [22]), and the statistics from 1988 to 2017 presented herein, with floods, storms, and droughts placed as the most frequent and damaging risks. The differences between the perceptions and the reality are attributed to (a) the small sample (and 75% of them from flood-prone countries Brazil, Paraguay, Uruguay, Argentina, and Venezuela), and (b) actually most extreme temperatures are cold waves instead of heat waves, which is likely due to more direct impacts of cold.

Media Literacy is perceived as a critical strategy for improving climate literacy. Media influence public awareness and opinion on climate change [52,55,56] and shape public understanding of scientific issues being the interface between science production and the lay public knowledge [57,58].

There is a widespread lack of confidence in the available information, preparedness, and actions on EWEs, the health systems and government’s DRR measures (ranging from 70% to 86%). However, some respondents have confidence in their national structures (e.g., Paraguay) which, despite its relatively lousy vulnerability indices, is in line with (i) the Framework Law on Climate Change (see excerpts of the participants in Section 3.2), (ii) the human impacts due to EWEs (where Paraguay looks better than expected); and (iii) its relatively good Legatum Prosperity Health Index-LPHI (4th).

Several respondents to the online survey believe that policy actions should focus on improving/promoting the health capacities, including (i) research; (ii) partnerships and stakeholders’ participation; (iii) Information systems, climate-risk assessment, and early warning systems; (iv) best practices; (v) climate-health nexus research communication at several levels; and(vi) climate research. 

Quite surprisingly, the fundamental causes of poverty and vulnerability were not prioritised, which could be explained by the belief that climate adaptation and disaster risk reduction focus on (government) actions, likely because the causes need strong long-term commitment and investments to be solved.

Finally, it is worth mentioning that discourse aligns with new ways to deal with this global climate challenge, namely climate-resilient pathways. Adaptation and sustainable development facing new paradigms to deal with the obstacles CSDs pose to the Health system [50], to global partnerships (see excerpts of the participants in Section 3.2) and promote research [59].

### 4.3. Adaptation Strategies to cope with Extreme Weather Events

Addressing the causes of vulnerability is a prerequisite for sustainability in managing the Risks of Extreme Events and Disasters to Advance Climate Change (and variability) Adaptation [3], and with the Sustainable Development Goal (SGD) 13 (Climate Action), which proposes to strengthen resilience and adaptive capacity to climate-related hazards [18,60]. Therefore, the authors stress the importance of improving the implementation of the following adaptation strategies to building resilience against climate-sensitive disasters in human wellbeing and health.

LA countries should develop concerted efforts to identify communities and vulnerable groups whose wellbeing and health is increasingly at risk [13], because healthy, sustainable communities are built on the foundations of both healthy human populations and healthy natural ecosystems [24]. To get this goal some of the needed actions are (i) fostering education coupled with broad public information campaigns for undertaking measures to help to address health and social inequalities resulted from climate-risks [61]; (ii) improving healthcare facilities for handling increased patient volume resulting from EWEs [60,61,62]; and, (iii) developing National Adaptation Plans-NAPs [2,17,37,38].

An integrated approach to addressing health vulnerability to climate, risk assessment, and climate-disaster preparedness and response [1,5,17,50] should include: (i) joining resource mobilisation for discussing climate-risks and extreme events [5,17]; (ii) linking DRR and Climate Change Adaptation (CCA) for addressing the human health vulnerability to and the risks of CSDs [5,63,64]; and, (iii) developing integrated forecasting, early-warning, and climate-related risk-mapping systems of extreme weather events [1,17,37,50,52,53,54,65] and partnerships among diverse stakeholders [61], e.g., between meteorological and health departments [62] and with partner agencies within the UN system [10].

Structural long-term adaptation strategies should include the increase in public health expenditures [18], universal health coverage, and alleviation of poverty [18,50,66,67].

### 4.4. Potential Limitations of the Paper

It is inevitable that there are some possible weaknesses because of the underlying assumptions, the selected indices, and relationships that are presented in this paper. Hence, its applicability and the main potential limitations are addressed. Firstly, this paper focused on the direct and short-term human impacts of extreme events and their associated climate-disasters—regardless of climate change but assumed to be triggered by it. However, the indirect effects on the environment wellbeing (e.g., the degradation of ecosystems and water resources, the proliferation of vector-borne diseases, damages to infrastructure, and human habitat), are crucial human wellbeing and health factors in the medium-term [18,20,24,68]. Similarly, the medium and long-term impacts of droughts (e.g., adequate access to sanitation, people displacement), not addressed herein, have been pointed out for the world [1] and Latin America in particular [18,19,20,44]. Secondly, the emphasis is given to total death and affected people, which is not the only human adverse effects, but likely the most important ones. Thirdly, aggregated country-level impacts do not allow for discriminating the many relevant geographical, local, socioeconomic, and gender issues. However, keeping in mind that the poor, elderly, women and children, and those living in vulnerable climatic and geographic sites, are differentially impacted, aggregated studies are useful for cross-comparison research. Despite the above shortcomings, the authors understand the analysis that is presented here is still considered valuable and a base for future more detailed investigation. 

## 5. Conclusions

Regarding the research problem focused on which are the leading causes of the impacts of extreme weather events on human wellbeing and health impacts in Latin American countries, the presented results suggest that they are mainly related to the socio-economic and human development, the geographic determinants of health vulnerability to climate, and the descriptors of health disparities. Nevertheless, mostly uncontrollable external climate drivers of impacts and exposure are a significant factor in the risk-prone countries.

The key findings for the implementation of promising structural adaptation measures and specific actions to reduce the human vulnerability and impacts, are as follows.

The countries with the fewer direct impacts on human wellbeing are the ones with the best human, socio-economic, and vulnerability indices, the highest per capita expenditures in Public Health, particularly those with the highest public spending, namely Chile, Uruguay, Costa Rica, Panama, Argentina, Paraguay, Brazil, and Mexico.

However, despite the relatively high health investments (regarding their GDP) of highly exposed countries, such as Honduras and Nicaragua, the combination of the frequent occurrence of extreme weather events, high climatic and geographical exposure, and poor development, determine their very high (in absolute and comparative terms) vulnerability and direct human impacts.

Various studies have outlined sets of adaptation measures, which may assist countries in Latin America and elsewhere to handle the adverse impacts of climate-sensitive disasters on human wellbeing and health. Based on the results that are presented in this paper and the literature the authors believe that the most cost-effective measures and strategies are the following:

The improvement of public awareness, integrated Observational-Forecasting-Modelling-Mapping, and Early Warning System, and the Preparedness and Response infrastructure, logistics, and management (short-term measure).

The increase in Health Expenditures focused on the reduction of disparities (medium-term measure).

The achievement of the Sustainable Development Goals—notably reducing poverty levels (long-term strategy).

Future research directions should include in-depth analysis of multi-variable quantitative relationships between all of the determinant factors of health vulnerability to climate, and direct and indirect impacts on human wellbeing and their life-supporting systems, as well as further assessments of the barriers, opportunities, and actions that are related to the effective implementation of linked climate adaptation and disaster risk reduction.

## Figures and Tables

**Table 1:** The geographical and climatic setting of extreme weather events.

Country (Region)	Area Mkm^2^	Pop Minh.	Main Climate Köppen Class. Types	Main Extreme Weather Events (EWEs)
Argentina (SA)	2.7	44	Temperate humid subtropical (Cfa)	Riverine floods, Storms, Wildfires, ext. (cold) temperatures, storm surges
Bolivia (SA)	1.1	11	Dry cold semi-arid (BSk)	Drought, landslides, wildfires, ext. temperatures (cold; heat wave)
Brazil (SA)	8.3	209	Tropical wet (Aw)	Riverine Floods; droughts; landslides, ext. (cold) temperatures
Chile (SA)	0.7	18	Temperate warm summer (Csb)	Riverine floods, wildfires, landslides, ext. (cold) temperatures
Colombia (SA)	1.1	49	Tropical rainforest (Af)	Riverine floods, landslides
Costa Rica (CA)	0.51	5	Tropical wet (Aw)	Storms, riverine floods, wildfires
Ecuador (SA)	0.25	17	Tropical wet (Aw)	Riverine floods, droughts, landslides, sea-floods
El Salvador (CA)	0.02	6	Tropical wet (Aw)	Storms, droughts, riverine floods
Guatemala (CA)	0.11	17	Tropical wet (Aw)	Droughts, storms, riverine floods, wildfires
Honduras (CA)	0.11	9	Tropical wet (Aw)	Droughts, storms, riverine floods, wildfires
Mexico	1.9	129	Hot semi-arid (BSh)	Storms, riverine floods, droughts, ext. (cold) temperatures
Nicaragua (CA)	0.12	6	Tropical wet (Aw)	Storms, riverine floods, droughts, wildfires
Panama (CA)	0.74	4	Tropical monsoon (Am)	Riverine floods, storms
Paraguay (SA)	0.4	7	Temperate humid subtropical (Cfa)	Riverine floods, droughts, wildfires, ext. (cold) temperatures
Peru (SA)	1.3	32	Oceanic (Cfb)	Riverine floods, ext. (cold) temperatures, droughts, storms, landslides
Uruguay (SA)	0.18	3.4	Temperate humid subtropical (Cfa)	Riverine floods, storms, ext. (cold) temperatures, storm surges
Venezuela (SA)	0.9	32	Tropical wet (Aw)	Riverine floods, landslides

**Table 2:** Development and Vulnerability status. Classed (1–5) ranked sectoral vulnerability indicators. Aggregated Vulnerability (CRV) and Adaptability (CRA).

	Human and Socioeconomic Development Poverty (Pov)			ND-Gain Index (Discrete Values)						
				Discrete Vulnerability Indices and Aggregated Vulnerability (CRV) and Adaptability (CRA)						
Country	Human 2016		Socio-Economic 2017	PHV	WV	HHV	E	S	Classed Ranked CRV and CRA. The Highest the Less Vulnerable/Most Adaptable	
	HDI 2016 Class	Pov (%) 2016	Per Capita GNI							
Argentina	Very High	30	High	2	2	2.5	3	1	15	15
Bolivia	Med	38	Lower-Middle	4.5	4	3.5	2.5	2.5	2	2
Brazil	High	13	Upper-Middle	2.5	1.5	2.5	4	1	12	15
Chile	Very High	11	High	1.5	2	2.5	1.5	1	17	17
Colombia	High	28	Upper-Middle	3.5	3.5	3	4	1	8	10
Costa Rica	High	20	Upper-Middle	1.5	2	3.5	2.5	2	12	15
Ecuador	High	23	Upper-Middle	3.5	2.5	4	4.5	2	4	8
El Salvador	Med	33	Lower-Middle	4.5	3.5	3.5	3	3	1	5
Guatemala	Med	59	Lower-Middle	4.5	2.5	3.5	3	3.5	2	8
Honduras	Med	66	Lower-Middle	4.5	2	3.5	2.5	3	5	2
Mexico	High	20	Upper-Middle	2	3	2	3	1	14	12
Nicaragua	Med	30	Lower-Middle	4.5	1	3.5	3.5	3	5	5
Panama	High	22	Upper-Middle	4	1	3	2.5	2.5	10	12
Paraguay	Med	29	Upper-Middle	2.5	5	3.5	2	1.5	9	10
Peru	High	21	Upper-Middle	4	3.5	4	2.5	1.5	5	5
Uruguay	High	9	High	2	1.5	4	2.5	2	11	16
Venezuela	High	32	Upper-Middle	3.5	1	2.5	2	1	16	8

**Table 3:** Gross domestic product (GDP) Per Capita, and Public and Private Expenditures in Health. The authors calculated ranks and Per Capita expenditures from reference [18].

Country	GDP (2014–2015)	Expenditures in Health (% of Country’s GDP)					
	Per Capita (Current US$, 2014–2015)	Public	Private	Total	Regional Rank	Per Capita	Regional Rank
Argentina	12,450	2.7	2.1	4.8	17	598	8
Bolivia	3000	4.6	1.8	6.4	13	192	16
Brazil	9900	3.8	4.5	8.3	7	822	5
Chile	14,100	3.9	3.9	7.8	9	1100	2
Colombia	7140	5.4	1.8	7.2	10	514	10
Costa Rica	10,400	6.8	2.6	9.4	2	978	3
Ecuador	6030	4.5	4.7	9.2	3	555	9
El Salvador	3800	4.5	2.3	6.8	11	258	13
Guatemala	3590	2.3	2.9	6.2	14	223	14
Honduras	2280	4.4	4.3	8.7	5	198	15
Mexico	9710	3.5	3.0	6.5	12	631	6
Nicaragua	1940	5.1	3.9	9.0	4	175	17
Panama	11,800	5.9	2.2	8.1	8	962	4
Paraguay	4190	4.5	5.3	9.8	1	411	11
Perú	6130	3.3	2.2	5.5	15	337	12
Uruguay	15,720	6.1	2.5	8.6	6	1352	1
Venezuela	11,780	1.5	3.7	5.2	16	613	7
Median	7140	4.5	2.9	7.8		555	

**Table 4:** Some selected descriptors of climate-risks, extreme events occurrence, and geographical exposure.

Country	GNI Per Capita (PPP) 2017 [39]	HDI 2016 [32]	CRI-2018 (1997–2016) [30]		LPHI 2017 [33]	EM-DAT Statistics 2018 [29]		Freq. Per Year	Geographical Exposure Classed as Pop. Density (PD) 1: Very Low 5: Very High
	US$ × 1000	Value/Reg. Rank	Reg. Rank	Fatal. Per M inh/Ranked Class	Reg Rank	Nb of Extreme Events. Time-Horizons: A: 1988–2017, B: 1997–2016		Freq. over A Trend From A to B	
						A	B		Classed PD
Argentina	20.3	0.83/2	5	0.7/1	6	76	57	2.5 ↗	1
Bolivia	7.3	0.67/14	13	0.45/13	17	66	48	2.2 ↗	2.5
Brazil	15.2	0.75/8	4	0.8/3	10	151	101	3.4 ≈	1
Chile	23.2	0.85/1	3	0.06/2	3	58	42	1.9 ↗	2
Colombia	14.2	0.73/11	10	2.4/9	7	101	67	1.9 ≈	3
Costa Rica	17.3	0.78/5	1	1.4/5	1	40	26	1.3 ≈	4
Ecuador	11.3	0.74 /9	9	2.9/11	9	44	28	1.5 ≈	4
El Salvador	7.5	0.68/13	14	5.3/14	8	46	28	1.5 ≈	5
Guatemala	8.0	0.64/16	15	7.2/15	16	67	48	2.2 ≈	4.5
Honduras	4.6	0.63/17	17	43/17	12	63	38	2.1 ↘	5
Mexico	17.7	0.76/7	11	1.3/4	5	169	126	4.2 ≈	2
Nicaragua	5.7	0.65/15	16	29.6/16	14	55	36	1.8 ≈	5
Panama	21.9	0.79/4	2	2.8/10	11	41	32	1.4 ↗	4
Paraguay	9.2	0.69/12	12	0.15/6	4	42	29	1.4 ≈	3
Perú	12.9	0.74/9	7	3.9/12	13	90	59	2.0 ≈	3
Uruguay	21.9	0.80/3	6	2/6	2	26	22	0.9 ↗	3.5
Venezuela	NA	0.77/6	8	2/8	15	40	26	1.3 ≈	1.5

↗: Increase; ≈ equal or similar; ↘: decrease.

**Table 5:** Occurrence and type of extreme weather/climatic disasters among the top-ten Natural Disasters and Total Affected People at country-level.

Country	TAP Nb of Top-Ten Extreme Weather Events							
	(Million People) Due to Top-Ten Events	Types of EWEs					TAP (%)	
		Rank	Nb of EWEs	Floods	Storms	Droughts	Landslides	Country-Level
Argentina	6.6	5th	10	10	0	0	0	15
Bolivia	2.7	8th	10	7	0	3	0	25
Brazil	50.5	1st	9	4	0	5	0	24
Chile	0.9	13th	7	6	0	0	1	5
Colombia	9.1	4th	9	9	0	0	0	18
Costa Rica	1.3	12st	9	4	5	0	0	26
Ecuador	0.7	15th	5	2	0	1	1	4
El Salvador	1.7	11th	7	1	3	2	0	28
Guatemala	5.9	7th	9	2	3	4	0	35
Honduras	6.0	6th	10	3	3	4	0	67
Mexico	10.7	3rd	10	3	6	1	0	8
Nicaragua	2.7	8th	9	2	4	3	0	45
Panama	0.13	17th	9	7	2	0	0	26
Paraguay	2.6	10th	10	5	0	3	0	18
Perú	12.3	2nd	9	3	1	2	0	38
Uruguay	0.19	16th	10	10	0	0	0	5
Venezuela	0.9	13th	10	9	0	0	0	3
Total			152	87	27	28	2	Median:
%			89	57	18	18	1	24

**Table 6:** Participants’ responses to the online survey.

**Question/Statement**	**The Extent to Which the Participants Agree with the Statement (in %)**				
	**Strongly Agree**	**Agree**	**Neither Agree nor Disagree**	**Disagree**	**Strongly Disagree**
“There is a connection between climate change and health.”	86	12	2	0	0
“There is enough information for the public about the health impacts of extreme events in my country.”	2	16	12	48	22
“I think the health system of my country is well-prepared and equipped to face health impacts from weather extremes and sea level rise.”	2	4	12	50	32
“I think the government of my country is doing enough to deal with the health impacts from weather extremes and sea level rise.”	0	4	10	46	40
“Is there anything else that you suggest could be done to ensure to protect human health and wellbeing from the impacts of extreme events?”	Yes	No	I don’t know		
	82	6	12		
*What could be done to ensure to protect human health and wellbeing from the impacts of extreme events?*					
**Suggestions made to ensure to protect human health and well-being from the impacts of extreme events**					
**Suggestion**	**Example**				**%**
1. Health capabilities	Health capacities; Promoting Health; Epidemiology of the identified priority vector-borne zoonotic diseases				16
2. Climate Literacy	Climate Change Education; Information and Media; Training and Lifelong Learning				16
3. Information systems in a changing climate					13
4. Early Warning Systems					12
5. Risk Assessment					6
6. Management and communication					6
7. Preparedness					4
8. Effective Implementation of National/Local Adaptation Plans (NAPs/LAPs)	Sectoral strategies (namely Agriculture, Health, and Economy)				4
9. Climate Change Research and Capacity Building					4
10. Partnerships and participation	Stakeholders and partnerships. Participation in building resilience in a changing climate				4
11. Others					15

## Supplementary Materials

The following is available online at http://www.mdpi.com/1660-4601/15/9/1802/s1, Table S1: Online Survey Responses.
