# Supplementary material for: An Assessment of the Relationships between Extreme Weather Events, Vulnerability, and the Impacts on Human Wellbeing in Latin America
Source: Int J Environ Res Public Health. 2018 Aug 21;15(9):1802. doi: 10.3390/ijerph15091802 (PMC6163949; doi:10.3390/ijerph15091802)
Supplement: Supplementary file 1 [file ijerph-15-01802-s001.pdf]

_Answer ID	Resume-Code	Start	Date and time	Participation s	1. 1. What is y	2. 2. Please in
41315970	516df5b	13.12.2017 19	13.12.2017 20	participated a	male	31 - 50 years
41326034	41c8458	14.12.2017 02	14.12.2017 02	participated a	female	20 - 30 years
41326530	fe3b624	14.12.2017 04	14.12.2017 04	participated a	male	51 - 70 years
41336105	47f24a0	14.12.2017 11	14.12.2017 11	participated b	female	51 - 70 years
41351979	aa8af74	14.12.2017 17	14.12.2017 17	participated a	male	31 - 50 years
41352755	74c62ef	14.12.2017 17	14.12.2017 18	participated a	male	51 - 70 years
41352795	c15a429	14.12.2017 17	14.12.2017 18	participated a	male	above 70 year
41353727	4bd172e	14.12.2017 18	14.12.2017 18	participated a	male	51 - 70 years
41353810	f89c69f	14.12.2017 18	14.12.2017 18	participated a	male	31 - 50 years
41353959	aec24f3	14.12.2017 18	14.12.2017 18	participated a	female	31 - 50 years
41354763	a1e70b5	14.12.2017 18	14.12.2017 18	participated a	female	31 - 50 years
41356289	78d13b9	14.12.2017 19	14.12.2017 19	participated a	male	51 - 70 years
41357521	c932aca	14.12.2017 20	14.12.2017 20	participated a	male	51 - 70 years
41373550	9d710d4	15.12.2017 11	15.12.2017 11	participated a	female	31 - 50 years
41375568	83e2a00	15.12.2017 12	15.12.2017 12	participated a	female	31 - 50 years
41377448	72eb810	15.12.2017 13	16.12.2017 21	participated a	female	51 - 70 years
41394013	be7a4ac	15.12.2017 22	15.12.2017 22	participated a	female	31 - 50 years
41410861	98654af	16.12.2017 20	16.12.2017 21	participated a	male	above 70 year
41424783	4d01fa0	17.12.2017 18	17.12.2017 19	participated a	female	31 - 50 years
41425418	d4c0694	17.12.2017 19	17.12.2017 19	participated a	female	31 - 50 years
41431756	9e868c4	17.12.2017 23	17.12.2017 23	participated a	female	51 - 70 years
41441320	b7af51c	18.12.2017 11	18.12.2017 11	participated a	female	31 - 50 years
41447794	79c79b8	18.12.2017 14	18.12.2017 14	participated a	female	51 - 70 years
41448424	6da4b57	18.12.2017 14	18.12.2017 14	participated b	female	31 - 50 years
41450060	88c42ae	18.12.2017 14	18.12.2017 14	participated b	female	20 - 30 years
41451001	d9bad6f	18.12.2017 15	18.12.2017 15	participated a	female	51 - 70 years
41452805	cadfdad	18.12.2017 15	18.12.2017 15	participated a	female	51 - 70 years
41455392	eacd34f	18.12.2017 16	18.12.2017 17	participated a	male	20 - 30 years
41457810	a113d73	18.12.2017 17	18.12.2017 17	participated a	female	31 - 50 years
41458447	1483b01	18.12.2017 15	18.12.2017 21	participated a	male	51 - 70 years
41459200	28c954e	18.12.2017 18	18.12.2017 18	participated a	female	20 - 30 years
41462899	3d89b99	18.12.2017 19	18.12.2017 20	participated a	female	31 - 50 years
41466185	a5cd3f0	18.12.2017 21	18.12.2017 21	participated a	male	20 - 30 years
41471189	862ea88	19.12.2017 00	19.12.2017 00	participated a	female	51 - 70 years
41627916	a283d4f	26.12.2017 13	26.12.2017 14	participated b	male	31 - 50 years
41639996	decd217	27.12.2017 11	27.12.2017 12	participated a	male	31 - 50 years
41644145	f9e9a1d	27.12.2017 14	27.12.2017 15	participated a	female	31 - 50 years
41646706	dbbe4b0	27.12.2017 16	27.12.2017 17	participated a	male	51 - 70 years
41647976	96101a4	27.12.2017 17	27.12.2017 17	participated a	male	51 - 70 years
41653766	122f294	27.12.2017 22	27.12.2017 23	participated a	male	51 - 70 years
41655950	9a128b6	28.12.2017 05	28.12.2017 05	participated a	male	31 - 50 years
41668495	ae107ce	28.12.2017 18	28.12.2017 18	participated a	male	51 - 70 years
41681433	2be1eeb	29.12.2017 12	29.12.2017 12	participated a	female	31 - 50 years
41682907	1f4f673	29.12.2017 13	29.12.2017 13	participated a	male	51 - 70 years
41687533	93b98f1	29.12.2017 17	29.12.2017 20	participated a	male	above 70 year
41691899	6a79fd5	29.12.2017 22	29.12.2017 22	participated a	female	51 - 70 years

41693757	c9ae3b0	30.12.2017 02	30.12.2017 02	participated a	female	51 - 70 years
41708783	cb05bf5	31.12.2017 12	31.12.2017 12	participated a	male	51 - 70 years
41709119	b8961fa	31.12.2017 13	31.12.2017 13	participated a	female	51 - 70 years
41709583	96b0ded	31.12.2017 13	31.12.2017 14	participated a	male	above 70 year
41741403	bce441d	02.01.2018 22	02.01.2018 22	participated a	female	51 - 70 years
41757995	4b93ac3	03.01.2018 16	03.01.2018 16	participated and completed		51 - 70 years
41790512	a862b11	04.01.2018 20	04.01.2018 20	participated a	female	51 - 70 years
41923054	79b09b6	09.01.2018 17	09.01.2018 20	participated a	female	31 - 50 years

3. 3. What is t	4. 4. What is y	5. 5. What is y	6. 6. Please cc	7. 7. What are	storms	storm surges
PhD	Portugal	academia	strongly agree			
Master's degr	Brazil	academia	agree		1	
PhD	Ecuador	academia	strongly agree			
PhD	Brazil	public service				
PhD	Uk	academia	strongly agree			
PhD	Mexico	academia	strongly agree		1	1
PhD	mexico	academia	strongly agree		1	
Bachelor's deg	USA	industry	strongly agree		1	1
PhD	Argentina	academia	strongly agree		1	
Master's degr	Colombia	NGO	strongly agree		1	
PhD	UK	academia	strongly agree		1	1
PhD	Mexico	academia	strongly agree			
DEA de Iii cycl	Chile	public service	strongly agree			
PhD	Brazil	academia	neither agree or disagree		1	
Pos Doc	Brasil	academia	strongly agree		1	
PhD	Brasil	academia	strongly agree			
PhD	Guatemala	academia	strongly agree			
Master's degr	AsunciÃ³n - Pa	academia	strongly agree			
Master's degr	paraguay	academia	strongly agree		1	
Master's degr	paraguay	academia	strongly agree		1	
Master's degr	uruguay	public service	agree			1
PhD	Brazil	academia	strongly agree		1	
PhD	Brazil	public service	agree		1	
Master's degr	Brazil	academia				
PhD	URUGUAY	public service	strongly agree			
Master's degr	Brazil	academia	strongly agree		1	
Diploma / Sec	Brazil	Student	agree		1	
Master's degr	Brasil	academia	strongly agree		1	
Master's degr	Bolivia	Free lance cor	strongly agree		1	
Master's degr	Brazil	academia	strongly agree		1	1
PhD	Argentina	public service	agree			
Bachelor's deg	Brasil	academia	strongly agree		1	1
Master's degr	Mexico	academia	strongly agree		1	
Master's degr	Colombia	industry				
Bachelor's deg	Brasil	academia	strongly agree			
Master's degr	Nicaragua	academia	strongly agree		1	
PhD	BRAZIL	academia	strongly agree		1	
PhD	Brazil	academia	agree		1	
Master's degr	Argentina	public service	strongly agree			
Master's degr	Brazil	academia	strongly agree			
PhD	Uruguay	academia	strongly agree		1	1
Master's degr	Venezuela	academia	strongly agree			1
PhD	Venezuela	academia	strongly agree			
PhD	Venezuela	academia	strongly agree		1	
PhD	Venezuela	academia	strongly agree			

PhD	Venezuela	academia	strongly agree			
PhD	Paraguay	Civil Society O	strongly agree		1	
PhD	Uruguay	academia	strongly agree			1
Master's degr	Paraguay	academia	strongly agree			
Master's degr	Venezuela	academia	strongly agree			
PhD	Venezuela	academia	strongly agree		1	
PhD	BOLIVIA	academia	strongly agree		1	
Bachelor's deg	Mexico	Consultancy	strongly agree		1	

droughts	heat waves	fires	inundation an	Text field	8. 8. Please cc	9. 8.1. If you (:
1	1	1			disagree	
1			1		disagree	
			1		agree	
	1		1		agree	
	1	1		hurricans	strongly disagree	
	1		1	earthquakes	disagree	
1	1	1	1		agree	
1		1	1		disagree	
1	1	1	1		strongly disagree	
	1		1		neither agree or disagree	
1			1		neither agree or disagree	
1		1	1		disagree	
		1	1		neither agree or disagree	
1		1	1		agree	
1			1	deforestation	disagree	
				hurricanes	disagree	
1		1	1		agree	inundation an
1	1		1		strongly disagree	
1	1		1		disagree	
1	1		1		disagree	
			1		disagree	
	1		1		disagree	
1	1		1		disagree	
1	1	1	1	sea level rise	strongly disagree	
			1	Shallow landsl	disagree	
		1	1		strongly disagree	
1	1	1	1	Hail	strongly agree	Flood Mamor
1	1	1	1		disagree	
			1		disagree	
1			1	Landslides	disagree	
1	1	1	1		disagree	
			1		strongly disagree	
1	1	1	1	plagues and il	disagree	
1		1	1		agree	Midia reports
1		1	1		agree	
	1		1		strongly disagree	
1			1		disagree	
1		1	1		disagree	
1	1	1	1		strongly disagree	
1			1		disagree	
1	1	1	1	Outbreak of m	neither agree or disagree	
1		1	1		neither agree or disagree	

			1		disagree
1	1		1		agree
1	1	1	1		disagree
			1		neither agree or disagree
			1		strongly disagree
1	1	1	1		strongly disagree
1		1	1	LANDSLIDES	disagree
1			1	hurricanes	strongly disagree

10. 9. 'I think t	11. 10. 'I think	12. 10.1. If yo	13. 11. Is ther	14. 11.1. If 'yes', please provide examples:
disagree	disagree		yes	Efective Implementation of NAPs, NAS and
disagree	strongly disagree		I don't know	
disagree	neither agree or disagree		yes	More education at all leves
strongly agree	disagree		I don't know	
strongly disag	strongly disagree		yes	Clima change is related also with pollutants
strongly disag	strongly disagree		yes	eliminate the pattern of systematic discrim
disagree	disagree		yes	
disagree	disagree		yes	Early warning systems, Risk management ,
strongly disag	strongly disagree		yes	Education, awareness, public policies
agree	neither agree or disagree		no	
neither agree	disagree		yes	In my country there is a lack of informatior
disagree	strongly disagree		yes	We should develop effective and actualizec
disagree	neither agree or disagree		I don't know	
disagree	disagree		yes	AtravÃ©s da gestÃ£o de risco, do monitora
disagree	strongly disagree		yes	The development of public policies, massiv
disagree	disagree		yes	Proper information, vulnerability and risk a
neither agree	agree	Paraguay in th	yes	Prioritize to programs and projects and res
strongly disag	strongly disagree		yes	1- Research, 2- Public politics, 3- Engage all
strongly disag	disagree		yes	research, public politics, engage all stakeho
neither agree	disagree		I don't know	
disagree	disagree		yes	- Investiments in education, and lifelong lea
disagree	disagree		yes	Teach about this relation on elementary ar
disagree	neither agree or disagree		yes	Formulate a National Health Adaptation Pla
disagree	strongly disagree		yes	Campaigns with informations about the eff
strongly disag	strongly disagree		yes	More investment to researches and develo
disagree	disagree		no	
strongly disag	disagree		yes	Development local and national early warn
strongly disag	strongly disagree		yes	I believe that among the long-term measur
neither agree	disagree		yes	At national Level with Health Ministery we
strongly disag	strongly disagree		I don't know	
strongly disag	strongly disagree		yes	Improve early warning systems, , Develop r
strongly disag	strongly disagree		yes	One idea is to promote integrative health a
disagree	disagree		yes	Improve partnerships inside country, and S
disagree	disagree		yes	Change patterns of production and consur
disagree	strongly disagree		yes	Have a good health system
neither agree	disagree		yes	mitigation of climate change (not a priority
disagree	strongly disagree		I don't know	
agree	neither agree or disagree		yes	To improve modelling, observation, forecas
strongly disag	strongly disagree		yes	Inform the population, sensitize the popula
disagree	strongly disagree		yes	Capacity building in climate change and hea
disagree	disagree		yes	1. Promote at the country level programs t
strongly disag	disagree		no	

strongly disag	strongly disagree		yes	Developing early warning systems would b
disagree	disagree		yes	knowledge to the general citizenship, actio
strongly disag	strongly disagree		yes	The occurrence of diseases present in the r
neither agree	agree	There is a syst	yes	supports further research on the subject ar
disagree	disagree		yes	The Academy of Science.
strongly disag	strongly disagree		yes	If authorities already have the knowledgec
disagree	disagree		yes	Develop integral climate change health ada
disagree	disagree		yes	Act urgently to tackle the root of the probl

at the municipalities the LAPs (local adaptation plans) in the sectorial strategies (namely Health secto

s in aquifers. High concentration levels of pollutants are cummulated in dry seasons because there is
nination and exclusion and pretend that becuase people have a card they can get attention from instit

Emergency response and epidemiological data from events

n to the public about the subject
d channels of integrated scientific information (IPCC A5R), in accordance with platforms, mechanisms

amento e das aÃ§Ãµes de prevenÃ§Ã£o.
e disclosure in media, scholar education and researches.
ssessments that can transform into adequate planning and risk management.
earch in the field of climate change and also the application of technologies in agriculture, where clir
stake holders as community, universities, public sector, NGO.
olders

arning and infrastructure are the solution to improve the life quality of the population, in terms of pr
nd medical schools, Teach the population how to recognize vulnerable situations and/or sites, Warn t

an., Prepare Training Programs on Climate Change and Health for the health sector work teams., Forr
fects of climate change,, Effective fiscalization of buildings in risk areas
opment of tecnics to avoid the most of the social and economic damages.

ning sistems it should be artculated with hospital atedendances., Health systems in Bolivia have colla
es can be the implementation of disaster culture through basic education and increase nature protec
are attemping to identify high risk areas of flooding in argentina and we are atemping to associatte t

risk maps at local level, , Improve risk communication in vulnerable areas, and in vulnerable populatic

s the integration of healthy people living on a healthy planet. People usually have the concept of hea
outh-South and North-South cooperation., Promote best practices, increase the climate-health nexus
nption

in Argentina, quite the opposite), association of research and health experts with those in Brazil esp

st and early warning systems, , Improve research on vulnerable sites and people,, Improve preparedr
ation on this issue, educate the children and prepare them for an imminent change. Generate defens
alth issues a different level, Increase the adaptation programs or project at local level., More access t
o empower the community on the potential effects of climate change on health., 2. Develop informa

e an excellent resource, with widespread information to the society in a simple way so the informatic
n plans for emergencies
region due to the effects of climate change (increase in temperature and precipitation) looms over ou
nd use communications from it, by the responsible organisms

d about the areas in the contry that will be strongly impact by the climate change, they should began
aptation Plans
em in a global scale: human-induced climate change! In this, we all have a role to play, and we must p

or) , and promoting Climate Literacy to general publics, school publics/students (namely Public Healt

not high flow of groundwater. 30% of the people drinking wÃ¡ter of Wells and sinkholes in the South
tutions in the public system

 and tools of the UNFCC (APA21), Talanoa Dialogues (Fidji-Bonn COP 23), to all levels of the State, pri

nate change impacts the most impact and also in health and in the economy, because the conseque

revent diseases.
he population in advance on any abrupt change on the weather forecast, Provide the population list

nulate Environmental Health Indicators linked to climate change and the health status of the populat

psed under normal conditions, so management in emergency care should be improved.
ction programs.
he main factors with the most flood related diseases in Argentina. This prioirity areas could help to c

ons (children, women, elderly, disable people), , Improved epidemiological surveillance regarding dise

lth as an individual attribute. The XXI century needs the integrations os self-care, care for the other a
s research communication at several levels.

ecially for tropical diseases (Chagas etc...)

ness, readiness and coordination at all levels (National, Regional, Local)
e and prevention mechanisms to increase the resilience of social systems in the face of extreme clima
o financial support for project in climate change, Involve the medical community in the climate chang
tion campaigns in the mass media and through social networks., 3. Promote at all levels of formal ed

on can be reached to most of the population.

ur country., The health system is not prepared., The university is not training its professionals for thes

to provide credits to these potencially affected population to buy or to rent a second house, we have

play it to our fullest capacity.

h and Health higher education institutions) and segmented publics (namely the media).

of Mexico, in YucatÃ¡n., , Is neccessary programs to monitoring pollutants under sceneries of Climat

vates and Organisations, to make real conscience of the planetary dangers we are inmersed in.

nces of climate change have a direct impact on the economy.

of to do things in case of extreme events, Begin to consider those factors in land use policy

tion, including information related to the burden of diseases associated with climate change., Implem

dirve resourses, vaccines and other basic tools to more frequently affected regions, More informatior

eases associated to hidrometeorological events

and care for the whole planetary ecosystem. There is a need for a new ethos (neoethos) in our health

ate changes.
ge theme
ucation the topic of climate change, and especially on the issues associated with health., 4. Design pr

se contingencies., We are not implementing adequate information campaigns and awareness of the p

e the expierence of the traumatic floods in 1999 in Vargas, coastal area in venezuela, thousands peop

e Change, and Promotion Health Programs to face Climate Change and their impat to the Public Helat

ıent predictive models of the behavior of vector diseases and zoonoses, associated with climate chan

ı about prevention in media is needed.

professions, we need transform our paradigms of health.

ograms to increase the capacity of response in the area of health, of the official organizations before

ɔopulation about the risks we will have in the near future.

ɔle died, and thousand were displacement, the reconstruccion was very difficult and is still on going, a

th., , I would like to participate to development a educational program in my country, mainly at Maya

ge., Develop and implement a National Plan for prevention of diseases transmissible by vectors sensi

potential risks derived from climate change and extreme events.

ıt that time it was a surprising event, but right now with all the studies it is possible to prevent these

a RegiÃ³n st south of Mexico. We have evidence of high levels of pollutants in wÃ¡ter and their bioacc

itive to climate change., Develop and implement an early warning system for extreme temperature e

consequences.

:ummulation in the human being.

vents (heat and cold waves) and it is in execution in at least two departments of the country., To forr

nulate a diagnosis of the response capacity and the infrastructure of health care services and centers

[illegible] in the face of extreme weather-related events.
